# Biased Exposure–Health Effect Estimates from Selection in Cohort Studies: Are Environmental Studies at Particular Risk?

**DOI:** 10.1289/ehp.1408888

**Published:** 2015-05-08

**Authors:** Marc G. Weisskopf, David Sparrow, Howard Hu, Melinda C. Power

**Affiliations:** 1 Department of Epidemiology, and; 2 Department of Environmental Health, Harvard T.H. Chan School of Public Health, Boston, Massachusetts, USA; 3 Veterans Affairs Normative Aging Study, Veterans Affairs Boston Healthcare System, and Department of Medicine, Boston University School of Medicine, Boston, Massachusetts, USA; 4 Division of Epidemiology,; 5 Division of Global Health, and; 6 Division of Occupational and Environmental Health, Department of Medicine, Dalla Lana School of Public Health, University of Toronto, Toronto, Ontario, Canada; 7 Department of Epidemiology, Johns Hopkins Bloomberg School of Public Health, Baltimore, Maryland, USA

## Abstract

**Background:**

The process of creating a cohort or cohort substudy may induce misleading exposure–health effect associations through collider stratification bias (i.e., selection bias) or bias due to conditioning on an intermediate. Studies of environmental risk factors may be at particular risk.

**Objectives:**

We aimed to demonstrate how such biases of the exposure–health effect association arise and how one may mitigate them.

**Methods:**

We used directed acyclic graphs and the example of bone lead and mortality (all-cause, cardiovascular, and ischemic heart disease) among 835 white men in the Normative Aging Study (NAS) to illustrate potential bias related to recruitment into the NAS and the bone lead substudy. We then applied methods (adjustment, restriction, and inverse probability of attrition weighting) to mitigate these biases in analyses using Cox proportional hazards models to estimate adjusted hazard ratios (HRs) and 95% confidence intervals (CIs).

**Results:**

Analyses adjusted for age at bone lead measurement, smoking, and education among all men found HRs (95% CI) for the highest versus lowest tertile of patella lead of 1.34 (0.90, 2.00), 1.46 (0.86, 2.48), and 2.01 (0.86, 4.68) for all-cause, cardiovascular, and ischemic heart disease mortality, respectively. After applying methods to mitigate the biases, the HR (95% CI) among the 637 men analyzed were 1.86 (1.12, 3.09), 2.47 (1.23, 4.96), and 5.20 (1.61, 16.8), respectively.

**Conclusions:**

Careful attention to the underlying structure of the observed data is critical to identifying potential biases and methods to mitigate them. Understanding factors that influence initial study participation and study loss to follow-up is critical. Recruitment of population-based samples and enrolling participants at a younger age, before the potential onset of exposure-related health effects, can help reduce these potential pitfalls.

**Citation:**

Weisskopf MG, Sparrow D, Hu H, Power MC. 2015. Biased exposure–health effect estimates from selection in cohort studies: are environmental studies at particular risk? Environ Health Perspect 123:1113–1122; http://dx.doi.org/10.1289/ehp.1408888

## Introduction

Although randomized controlled trials (RCTs) are often conducted in a highly selected set of participants, exposure in such studies is unrelated to the selection process because exposures come after the randomization of selected participants. Therefore, it is reasonable to believe that this initial selection process does not induce biased exposure–health effect associations (i.e., associations that are different from the true total causal effect of the exposure on the outcome, in the absence of chance associations, in the source population that was sampled to obtain the study sample), although the findings of RCTs may lack generalizability (i.e., that the causal effect in the sampled population would not be the same as the causal effect in a different population). In observational studies, the initial selection process may result in biased exposure–health effect associations. Past or current exposure status may influence selection into the study or into substudies, either because exposure or correlates of the exposure are related to prespecified eligibility criteria or because they influence participant availability or willingness to participate. If both the exposure and the outcome or their correlates (including past exposure and outcome) are related to participation, a study’s exposure–health effect associations may not reflect the true total causal effect in the source population that was sampled to obtain the study sample, either because of selection bias (known as collider stratification bias in causal theory) (Hernán et al. 2004) or because the selection process is equivalent to conditioning on an intermediate between the exposure of interest and the outcome (Schisterman et al. 2009).

The impact of collider stratification bias is well recognized in the setting of case–control studies. By definition, participation is related to the outcome; if recruitment into a case–control study is related to the exposure of interest as well, the observed exposure–health effect association may not reflect the true causal effect in the sampled population (Hernán et al. 2004; Wacholder et al. 1992). Similarly, exposure–health effect associations in prospective cohort studies likewise may not reflect the true causal effect in the whole cohort (and by extension, the population from which the cohort was drawn) if both the exposure and outcome are related to loss to follow-up (Hernán et al. 2004). Perhaps less well appreciated is how the process of cohort formation can induce a similar problem. Structurally, this problem is the same as loss to follow-up. If the exposure and outcome, or their correlates, influence a person’s initial eligibility or participation, the resulting exposure–health effect association may not reflect the causal effect in the source population.

The impact of conditioning on an intermediate is also well recognized in the epidemiologic literature. Termed “overadjustment” by some, in simulation, the resultant bias can be substantial (Rothman et al. 2008; Schisterman et al. 2009). However, as with collider stratification bias, it is less well recognized that the cohort formation process may induce this bias in specific situations.

Many studies of environmental toxicant exposures are likely susceptible to bias of the exposure–health effect estimates attributable to the study or substudy selection process—including both collider stratification bias and bias due to conditioning on an intermediate—for two reasons: *a*) Environmental exposures are often related to socioeconomic status (SES), which is known to predict participation (de Graaf et al. 2000; Howe et al. 2013; Mein et al. 2012; Weuve 2013), and *b*) exposure to an environmental toxicant at one time point is often reasonably correlated with exposure at other time points, and prior exposures (and their consequences) may be related to participation. Another, perhaps simpler way to think of this relies on the fact that environmental exposures and their correlates (e.g., SES) are ubiquitous in space and time—the most susceptible individuals may not participate in studies given downstream consequences of past exposure or its correlates (potentially including the outcome of interest), leading to an underestimate of any adverse causal effects of exposure on a given health effect.

In this review, we examine the issue of biased exposure–health effect associations resulting from who participates in a study or substudy, and ways of reducing this bias in the analysis. To do this, we use an example of the association between cumulative lead exposure (as measured by lead concentration in bone) and mortality (total, all cardiovascular, and ischemic heart disease) in the Normative Aging Study (NAS), expanding on an earlier analysis (Weisskopf et al. 2009). NAS participants were originally enrolled in the 1960s and bone lead concentration was measured between 1991 and 1999 for a substudy of the health effects of lead exposure. Given the routes of lead exposure and cumulative nature of bone lead measures, measured bone lead concentration may include, and is almost certainly correlated with, lead exposures before cohort formation. Therefore, it is reasonable to think that *a*) unique prior correlates of lead exposure and mortality influence study participation at both study inception and recruitment into the lead substudy, resulting in collider stratification bias in the absence of analytical methods to mitigate this bias; and *b*) lead exposure–related health effects may influence study participation in both the original study and the lead substudy, resulting in bias from conditioning on an intermediate of the lead exposure–mortality association. To illustrate this, we describe how nonparticipation at each point of recruitment may bias the association between lead exposure and mortality using causal directed acyclic graphs (DAGs). We then use critical evaluation of the proposed causal structure to suggest methods by which we could mitigate this bias and apply these methods to illustrate the influence of such methods on study results.

## Methods

*Study population.* The NAS is a prospective cohort study of community-dwelling men initiated in 1963 (Bell et al. 1972). Eligibility criteria for entry into the NAS included having no history of treatment for hypertension; systolic blood pressure ≤ 140 mmHg; diastolic blood pressure ≤ 90 mmHg; and no other chronic conditions, including heart disease, diabetes mellitus, and cancer. At cohort inception, 2,280 participants between the ages of 21 and 80 years were recruited from the Greater Boston, Massachusetts, area, and since then, participants have come in every 3–5 years for in-person evaluations. Because the percentage of participants who are nonwhite (2%) or missing race (3%) is very low, we consider only the 2,159 white NAS participants. This research was approved by the Human Subjects Committees of the VA Boston Healthcare System, the Brigham and Women’s Hospital, and the Harvard T.H. Chan School of Public Health. Study participants provided informed consent at enrollment and at follow-up evaluations.

*Blood lead measurement.* Blood draws for blood lead concentration measurements were done at each regular NAS visit, starting in 1992. In all, there were 1,206 white NAS men with at least one measurement of lead in blood. For the analyses we used the first blood lead measurement for each individual, which were collected from 1992 through 2007. Blood was collected in special lead-free tubes containing EDTA and analyzed at ESA Laboratories (Chelmsford, MA) by Zeeman background-corrected flameless atomic absorption (graphite furnace) as previously described (Weisskopf et al. 2009).

*Bone lead measurements and substudy.* Between 1991 and 1999, a subset of NAS participants agreed to have their bone lead concentration measured to provide an estimate of cumulative past lead exposure over years to decades (Wilker et al. 2011) for a substudy of the health effects of lead exposure. Bone lead measurements were taken at both the patella and mid-tibial shaft with an ABIOMED K-shell X-Ray fluorescence (KXRF) instrument (ABIOMED, Danvers, MA) as described previously (Weisskopf et al. 2009). Units of lead concentration are in micrograms lead/gram bone mineral, and each measurement has an accompanying uncertainty related to background noise in the signal extraction procedure. We followed typical practice (Hu et al. 1996) and excluded measurements with estimated uncertainty beyond the typical range (> 10 and > 15 μg/g for tibia and patella, respectively) because this usually reflects excessive subject movement during the measurement. In total, 835 white NAS men had valid patella lead measurements, and 836 had valid tibia lead measurements.

*Mortality follow-up.* Most deaths of NAS participants are identified through next of kin or postal authorities. Additional deaths are identified via birthday cards and supplemental mailed questionnaires sent to the participants (when next of kin return them informing us of a death), as well as VA and Social Security Administration Death Master File searches. We considered deaths through March 2007 to be consistent with our earlier report (Weisskopf et al. 2009). Death certificates are obtained from the appropriate state health departments for deceased NAS participants and were reviewed by a board-certified cardiologist to assign cause of death according to the *International Classification of Diseases, 9th Revision* (ICD-9). On the basis of any cause listed on the death certificate (underlying or contributing), we classified deaths with ICD-9 codes 390 to 459 as attributable to cardiovascular disease, and ICD-9 codes 410 to 414 and 429.2 as attributable to ischemic heart disease.

*Causal directed acyclic graphs (DAGs).* Causal DAGs are a type of causal diagram that graphically represents the underlying causal relations between variables (both measured and unmeasured) in a given study setting. Such diagrams are useful in identifying key assumptions made about the causal structure of a problem and aid in identification of potential bias in estimating causal effects under a variety of analytic scenarios (Glymour et al. 2005, 2008; Hernán and Robins 2016).

**Figure 1:**
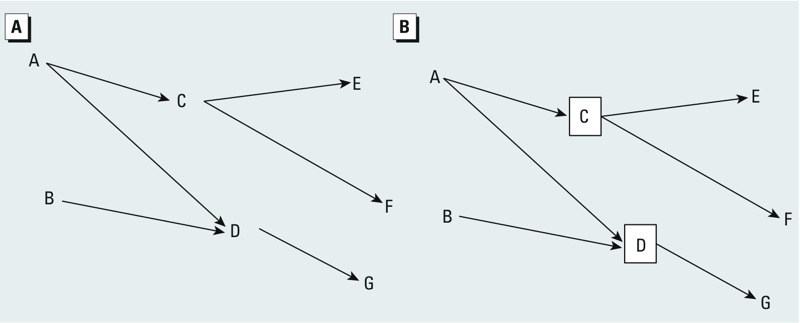
(*A*) Introduction to causal DAGs: A, B, C, D, E, F, G represent variables, or nodes, and directional arrows indicate causal relationships between these variables. A variable with two arrows pointing into it (a common effect of the two variables, e.g., D) is referred to as a collider. (*B*) Conditioning on a variable (either by restriction, stratification, or adjustment in a model) is indicated by drawing a box around the variable, as shown for variables C and D. See text for details on how DAGs indicate causal and noncausal associations between variables.

DAGs contain variables (also called nodes) and directional arrows between the variables (Figure 1). Arrows between any two variables denote that one variable causes the other (e.g., A causes C and C causes E in Figure 1A). Statistical associations between two variables are identified, with a few notable exceptions discussed below, as any connection through arrows (ignoring the direction of the arrows) between any two variables, including connections passing through other variables—this is known as a path. A statistical association between two variables is also a causal one if the path that emanates from the first variable (cause) and arrives at the other variable (effect) only travels in the direction of the arrows (e.g., A to F in Figure 1A), assuming the DAG is correctly constructed. Other paths that connect an exposure to the outcome, but include a portion that goes backwards along at least one arrow, indicate a statistical, but noncausal, association (e.g., D to C in Figure 1A). One limitation of DAGs is that, although they identify a potential source of bias, they do little to inform whether the magnitude of the bias is small or large, and the direction of bias is not always obvious.

Under some conditions variables connected by arrows are not statistically associated; in DAG terminology these conditions block a path. First, if there is a variable with two arrows that point into it (a common effect of two variables, known in DAG terminology as a collider, e.g., variable D in Figure 1A), the path for statistical association through that collider along these two arrows is blocked. Consequently, barring other paths, there will not be a statistical association between the two variables from which the arrows come (e.g., between variables A and B in Figure 1A).

Second, conditioning on a variable blocks statistical association that would otherwise go through that variable. In DAG graphical representation, a box drawn around a variable (e.g., variable C or D in Figure 1B) represents conditioning on that variable, which can be done by restriction, stratification, or regression adjustment. Thus, in Figure 1B, for example, there is no association between A and F after conditioning on C. However, there is one exception to this rule: Conditioning on a collider opens the path for statistical association through the collider along the two arrows into the collider (so there will be a statistical association between the two variables from which the arrows come, e.g., between A and B after conditioning on D in Figure 1B). In more formal language, conditioning on a collider makes marginally independent variables correlated within levels of the collider.

Finally, conditioning on a descendant of a variable (an effect of a variable) has effects similar to conditioning on the variable itself. For example, if you condition on variable F in Figure 1A (a descendant of C), the association between A and E will be partially blocked because you have partially conditioned on C. The degree to which conditioning on F conditions on C depends on the strength of the association between C and F. For example, adjusting for a weak effect of smoking (e.g., bad breath) would be insufficient to effectively condition on smoking behavior. In a similar manner, if you condition on variable G in Figure 1A (a descendant of the collider D), you partially open the path between A and B through D, which creates an association between A and B. Again, the degree to which that path is opened by conditioning on G depends on the strength of the association between D and G.

Bias of an exposure–outcome association occurs when there is any noncausal path on the DAG from the exposure to the outcome. In Figure 1A, there is no causal effect of D on F, yet there is a statistical association through the path D←A→C→F, indicating that an estimate of the causal association between D and F would be biased if the D←A→C→F path is not blocked (e.g., through conditioning on A or C). Similarly, in Figure 1B, there is an association between A and B (via the path A→D←B) in the absence of a causal effect, so we conclude our estimate of the causal effect of A on B (or vice versa) will be biased from a model that conditions on D. Such bias can result in an estimate of an exposure–outcome association that is stronger or weaker than the true causal effect, and has the potential to mute or even reverse the direction of the association relative to the true causal effect.

Bias can also occur when the selection process results in conditioning on an intermediate between the exposure and outcome of interest, because this produces an exposure–outcome estimate that does not reflect the causal effect of the exposure on the outcome in the source population. For example, in Figure 1B, we condition on C, an intermediate of the causal effect of A on E. If this conditioning was done by restricting the study sample to those in the source population who had a given level of C, then we would not see an association between A and E despite a causal effect of A on E in the source population. When one analyzes an entire population, or a representative sample of a population (i.e., a truly random sample of the entire population), then an exposure–outcome association found when conditioning on an intermediate can accurately estimate any effect of the exposure that is not through the intermediate on which one conditions, under certain assumptions (e.g., no confounding of the intermediate and outcome) (Schisterman et al. 2009). However, when conditioning on an intermediate is the result of selecting and analyzing only a subset of the original population (as is the case in our situation described below), then the exposure–outcome association may not be a valid estimate of either the total effect of the exposure on the outcome, or the portion of the effect operating through causal pathways that do not involve the intermediate variable.

**Figure 2:**
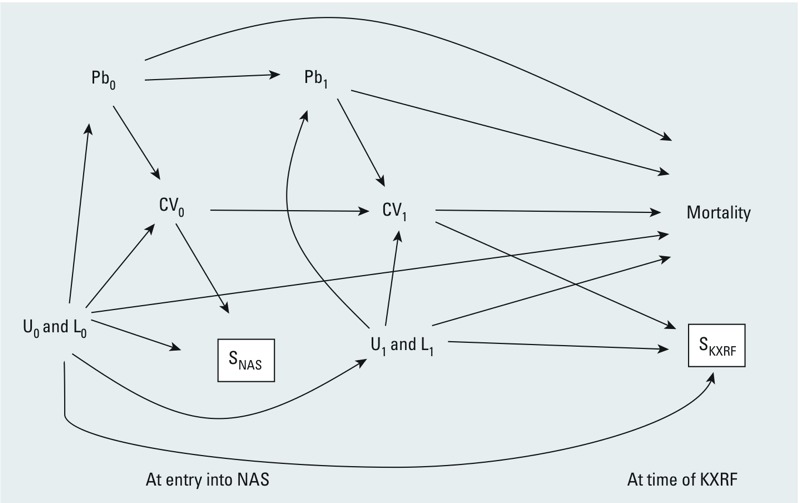
DAG representation of our assumptions about the structure of the data in the study, where we are interested in estimating the causal effects of cumulative lead exposure on mortality. See “Methods” for details. Abbreviations: CV, cardiovascular symptoms; L, measured variables; Pb, lead exposure/bone lead concentration; S_KXRF_, selection into the KXRF substudy; S_NAS_, selection into the NAS cohort; U, unmeasured variables. Subscripts 0 and 1 refer to the time of NAS recruitment and KXRF measurement, respectively. The U and L variables are separate variables, but the structure of arrows into and out of them are the same, and so for simplicity they are shown together.

*DAG representing our data.* The DAG shown in Figure 2 represents our assumptions about the structure of the causal relationships between lead, mortality, and related variables in our data. The subscripts refer to variables at entry into the NAS (0) and at the time of KXRF bone lead measurement (1). This DAG does not show all of the possible variables of interest individually—for simplicity, CV_0_ and CV_1_ represent clinically detectable cardiovascular disease or cardiovascular risk factors at times 0 and 1, L represents measured covariates, and U represents other unmeasured covariates. In addition, we combined U and L into a single node because the arrows into and out of the L and U variables have the same structure. Education is a good example of an L variable because it is known to affect study nonparticipation and loss to follow-up and is related to both health status and lead exposure. Other SES factors could be potential U variables. Study participation is denoted as S (for selection); two types are shown, S_NAS_ and S_KXRF_. The former indicates participation in the NAS cohort at its inception and the latter refers to participation in KXRF measurements. These two selection steps combine to determine the people that can actually be analyzed to examine the association between bone lead concentration and mortality. Both S_NAS_ and S_KXRF_ have boxes around them because restricting to the subset that entered the NAS and the subset of those who had KXRF measurements is a form of conditioning. The CV_0_→S_NAS_ and CV_1_→S_KXRF_ arrows are included because health status, including cardiovascular problems, is known to affect participation in epidemiologic studies (generally, the more healthy are more likely to initiate and maintain participation) (Alonso et al. 2009; Mein et al. 2012). In addition, observed cardiovascular health issues were part of the eligibility criteria at NAS entry (Bell et al. 1972). The U_0_&L_0_→S_NAS_, U_0_&L_0_→ S_KXRF_, and U_1_&L_1_→S_KXRF_ arrows are included because we are assuming some subset of the L (e.g., smoking, education) and U (e.g., other markers of SES, health status) variables affect study nonparticipation and loss to follow-up (generally, those with lower SES, those with worse health, and smokers are less likely to initiate and maintain participation), an assumption well supported by the literature (de Graaf et al. 2000; Howe et al. 2013; Mein et al. 2012). The U&L→Pb arrows are included because we are assuming some subset of the L and U variables (e.g., age, SES variables) are also causally related to lead exposure (e.g., older age and lower SES are associated with higher lead concentration) (Park et al. 2009). Similarly, the U&L→CV arrows indicate the assumption that some subset of the L and U (e.g., age, SES variables) are causally related to development of poor cardiovascular health (e.g., older age and lower SES are associated with more health problems). Pb_0_ and Pb_1_ represent summary measures of lead exposure at time 0 and time 1; however, we measure Pb only at time 1, so Pb_0_ is unobserved. However, we consider an effect emanating from Pb_0_ or Pb_1_ and terminating in mortality to represent a causal effect of Pb on mortality for the purposes of identifying potential bias in our DAG. This is a reasonable approach given that Pb_1_ and Pb_0_ are both measurements of cumulative lead exposure. Note that *a*) the observed Pb_1_ could include Pb_0_ exposure from before NAS entry, as represented by the arrow from Pb_0_ to Pb_1_ in Figure 2; and *b*) an individual’s exposure to lead is likely correlated over time (not depicted in the DAG). We wish to examine the total causal effect of Pb (Pb_0_ or Pb_1_) on mortality in the source population. This total causal effect includes all causal paths through or independent of other variables (e.g., CV), because we hypothesize that lead exposure could contribute to mortality both through and independent of induction of cardiovascular disease.

*Possible sources of bias.* Standard epidemiological analyses tend to focus exclusively on bias of exposure–health effect estimates from confounding. Both the L and U variables in our DAG introduce typical confounding bias because they are a common cause of the exposure and outcome under study: Statistical association between, for example, Pb_0_ and mortality occurs via a path that does not emanate from Pb_0_ (Pb_0_←L_0_→Mortality). However, assuming our DAG is correctly specified, the DAG reveals that there are two other potential sources of bias that arise as a result of who participated in the NAS study and KXRF substudy. Both may result in the analysis of a sample that is less likely to be susceptible to the adverse health effects of lead exposure than the general population, which would lead to an underestimate of the observed effect estimate of lead exposure on mortality. These are collider stratification bias and bias from conditioning on an intermediate.

**Figure 3:**
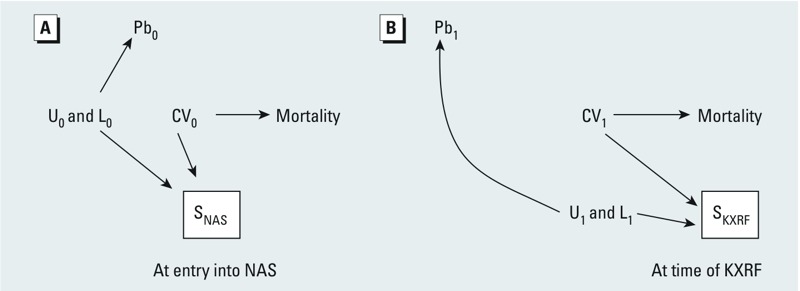
Illustration of selection bias (or collider stratification bias) (*A*) at the time of entry into the NAS and (*B*) due to loss to follow-up before KXRF lead measurements. See “Methods” for details. See Figure 2 for variable definitions.

*Collider stratification (selection) bias*. Collider stratification bias (i.e., selection bias) is a source of potential exposure–health effect estimate bias that is often not considered in analyses that are not case–control studies. In our study, if we ignore for the moment issues related to selection into the KXRF substudy and focus on selection into the NAS, we can consider the simplified DAG of Figure 3A, including only a portion of the Figure 2 DAG, that illustrates this problem. S_NAS_ is a collider on which we condition through the process of cohort formation. Therefore, statistical association between L_0_ and CV_0_ is induced along the path CV_0_→S_NAS_←L_0_ and Pb_0_ is connected to mortality via a path that does not emanate from Pb_0_ (Pb_0_←L_0_→S_NAS_←CV_0_→Mortality). This path is noncausal because it does not emanate from Pb_0_ (but goes backward along the arrow from Pb_0_), so analyses that ignore this may be biased. L_0_ could be replaced with U_0_ in the path above. This bias has exactly the same structure as collider stratification bias from loss to follow-up before selection into the KXRF substudy, which can be seen if we only consider the follow-up period after entry into the NAS (Figure 3B). Conditioning on S_KXRF_ opens the path CV_1_→S_KXRF_←L_1_ (or U_1_) and thereby we can observe an association between Pb_1_ and mortality in the absence of a causal effect of Pb_1_ on mortality.

**Figure 4:**
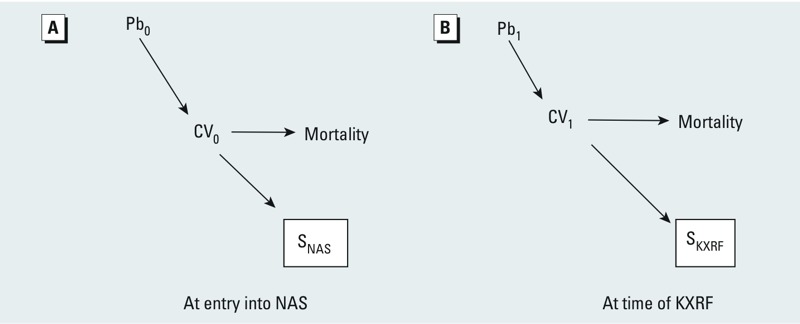
Illustration of bias due to conditioning on an intermediate (*A*) at the time of entry into the NAS and (*B*) due to loss to follow-up before KXRF lead measurements. See “Methods” for details. See Figure 2 for variable definitions.

*Bias from conditioning on an intermediate*. A second, often ignored, potential source of bias of exposure–health effect estimates is conditioning on an intermediate. Although this is more frequently recognized in terms of variables that lie on the causal path between an exposure and outcome, that this can occur as a result of selection processes is more often missed. This arises from the fact that conditioning on a descendant of a variable is akin to conditioning on the variable itself, especially if the association between the variable and its descendant is strong. Thus, in our study, if participation or selection into the NAS is at least partly driven by cardiovascular disease at the time of enrollment, then the participant selection process effectively conditions on CV_0_, a causal intermediate between lead exposure and mortality. This is illustrated in simplified form in Figure 4A. Conditioning on S_NAS_ blocks some of the association that goes from Pb_0_ to mortality through CV_0_, because conditioning on S_NAS_ partially conditions on CV_0_. We know this to be true at NAS inception, because absence of cardiovascular disease and hypertension were eligibility requirements; thus we expect downward bias of the lead–mortality effect estimate from an analysis that ignores this, masking the total true adverse effect of lead exposure on mortality in the source population from which we sampled to form the NAS. Similarly, we expect this problem to repeat at the time of selection into the KXRF substudy (Figure 4B) because health status is a known predictor of study participation and loss to follow-up (Alonso et al. 2009; Mein et al. 2012). However, the downward bias of the lead–mortality effect estimate at this stage may be less than at NAS entry because there were no explicit health related inclusion criteria for the KXRF substudy.

**Figure 5:**
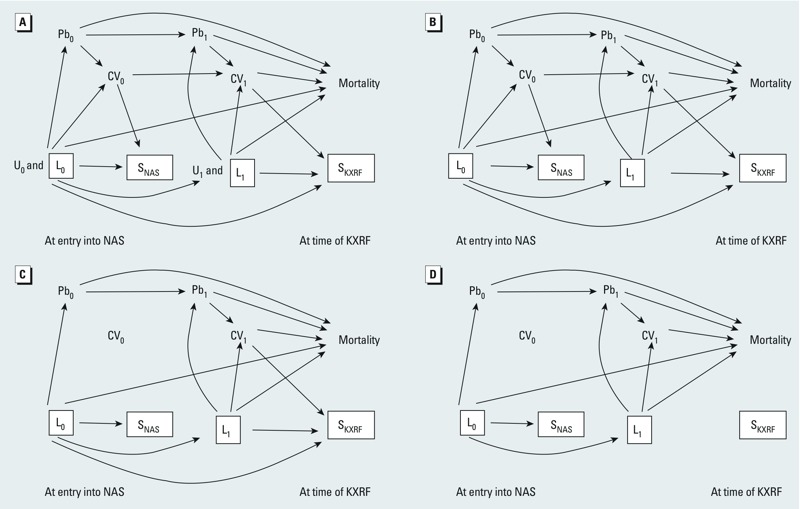
Demonstration of impact of efforts to alleviate bias due to collider stratification bias and conditioning on an intermediate (CV). (*A*) DAG reflecting structure of our data in the base analysis among white men adjusting for age, education, and smoking (Model 1). (*B*) DAG reflecting data structure after additional regression adjustment (model 2) under the assumption that we no longer have important uncontrolled variables (U), although we recognize that we cannot rule out such variables entirely. (*C*) DAG reflecting data structure after additionally restricting to those ≤ 45 years of age at NAS entry. (*D*) DAG reflecting data structure after additionally using IPW to account for loss to follow-up between cohort inception and KXRF among those ≤ 45 years of age at baseline. See “Methods” for details. See Figure 2 for variable definitions.

*Methods to account for bias.* Adjustment. Adjustment for relevant L variables assessed both at baseline and at the time of the KXRF substudy—standard practice in most epidemiological studies—should be sufficient to address biases of the lead–mortality effect estimates from both confounding and collider stratification biases introduced by these L variables (assuming adequate and appropriate measurement and parameterization of the L variables, and that the earlier lead exposure does not causally affect the later L variables, in which case conditioning on them would be conditioning on an intermediate). However, if there remain uncontrolled U variables as depicted in the DAG (Figure 5A), both confounding and collider stratification bias of the lead–mortality effect estimate will remain. (Adjusting for CV to block collider stratification bias is not possible because it induces conditioning on an intermediate of the effect of lead on mortality.) Adjusting for additional variables can help to remove some of the bias introduced by the uncontrolled U variables by effectively converting them to controlled L variables (in practice, U variables may actually be measured, but just not initially considered as potential confounders by the investigator)—for example, adjusting for other SES variables beyond education (Figure 5B). Such adjustment may also help reduce bias from yet other uncontrolled (U) SES variables to the extent that the controlled SES variables act as proxies for other uncontrolled (possibly unmeasured) SES variables.

Restriction. If we could eliminate the arrow between Pb_0_ and CV_0_ or CV_0_ and S_NAS_, the bias of the lead–mortality effect estimate from both collider stratification bias and conditioning on an intermediate at cohort recruitment would not exist. This could be done by restricting the analysis to those who were young enough at entry into the NAS (e.g. ≤ 45 years old) that cardiovascular disease–related health effects of lead exposure that affect participation would be relatively rare. In such a group, there are no arrows from Pb_0_ to CV_0_ or from CV_0_ to S_NAS_ because there are essentially no CV_0_ (Figure 5C). Removing the CV_0_ to S_NAS_ arrow also removes collider bias from conditioning on S_NAS_ whether that bias involves an L variable or a U variable, and so is more effective at removing this bias than simple adjustment for L variables.

Inverse probability weighting. Restriction based on age at entry into the NAS does not eliminate similar collider stratification bias or bias from conditioning on an intermediate resulting from selection into the KXRF substudy (Figure 5C). We cannot restrict to a group ≤ 45 years old at the time of KXRF because there are virtually none, given the age at NAS recruitment and time from that until the KXRF substudy. Instead, here we can use inverse probability of attrition weighting (IPW) (Hernán et al. 2000; Weuve et al. 2012) to alleviate the potential bias of the lead–mortality effect estimates from both collider stratification bias and conditioning on an intermediate resulting from selection into the KXRF substudy.

IPW uses information available for participants with and without KXRF measurements to weight observations from participants with a KXRF measurement, so that the weighted subpopulation is representative of all NAS participants who are alive at the time of the KXRF substudy. [It is possible to address attrition by death with this technique as well; however, this is controversial and particularly problematic for the current study, where the outcome is mortality—we refer readers to the work of others for further consideration of what to do with attrition due to death (Andersen and Keiding 2012; Chaix et al. 2012; Kurland et al. 2009; Lau et al. 2009; Tchetgen Tchetgen 2014; Tchetgen Tchetgen et al. 2012; Varadhan et al. 2014; Weuve et al. 2012)]. In this way the arrows into S_KXRF_ are removed (because the group is weighted to be representative of the whole living NAS population and, therefore, not a selected group of the original NAS study sample), thus eliminating the bias induced by conditioning on a collider or conditioning on the intermediate CV_1_ through conditioning on its descendant S_KXRF_ (Figure 5D). We could not use IPW to address the similar problem at NAS initiation because we have no data on those who did not participate in the NAS.

*Statistical analysis.* We used Cox proportional hazards modeling with age as the time metameter, to estimate adjusted hazard ratios (HRs) and 95% confidence intervals (CIs) for mortality in association with lead exposure. Participants contributed follow-up time from the date of their first blood or bone lead measurement to the date of death or last contact with the NAS. We adjusted for covariates by including the covariates in the model. We used missing indicators to account for missingness (< 5% for all variables except for mother’s occupation—21.4% missing—which likely reflected the mother’s not being in the workforce). We present DAGs for our base model (Figure 5A, model 1) and then illustrating the impact of layering on additional SES adjustment (Figure 5B, model 2), restriction (Figure 5C, model 3), and IPW (Figure 5D, model 4) to address issues of confounding, collider stratification bias, and conditioning on an intermediate.

Adjustment. Model 1 included age at blood or bone lead measurement and its square, smoking (never/former/current and linear pack-years), and education (less than high school, high school, technical school, some college, college graduate or more), represented by adding boxes around the L variables in Figure 5A. Model 2 also adjusted for additional markers of SES, including mother’s and father’s occupation (laborer, clerical, craftsman, manager, professional, other), mother’s and father’s education (grammar school, high school, college or more), occupation at NAS entry (categorized into broad job categories of the U.S. Bureau of the Census 2000 classification of private industry employees (U.S. Equal Employment Opportunity Commission 2013), and quintiles of salary at NAS entry. We assume that after this additional adjustment, we have no remaining influential uncontrolled confounders, and so have removed the U variables from Figure 5B. However, as in any epidemiological study one can never completely rule out unmeasured confounding.

Restriction. Model 3 includes model 2 covariate adjustment but only includes persons ≤ 45 years of age at NAS recruitment given that overt cardiovascular disease–related health effects of lead exposure that affect participation would be relatively rare at these younger ages. This eliminates all arrows into or from CV_0_ (Figure 5C).

Inverse probability of attrition weights. We used methods that have been described in detail elsewhere to create inverse probability of attrition weights (IPW) for non-death dropout after formation of the original NAS cohort (i.e., it will not address issues of selection into NAS) (Hernán et al. 2000; Kurland and Heagerty 2005; Power et al. 2013). Briefly, we used a single logistic regression model, with one record per study visit from inception through April 1999 (the last date of the bone lead measurements used in this study) to predict the probability of continuation in the study given that they were alive. Given the large number of possible predictors among the *a priori*–defined set of probable predictors (see Supplemental Material, Table S1) relative to the number of persons who dropped out of the study, we used forward selection to inform the variables included in the final models (see Supplemental Material, Table S2), as described in Supplemental Material, “Details of inverse probability weighting.” Unstabilized weights were calculated as the inverse of the final probability for each KXRF participant of remaining in the cohort at the time of KXRF lead assessment. Model 4 is the same as model 3, but includes this IPW weighting. In the DAG, IPW removes all arrows into S_KXRF_ (Figure 5D). The final DAG, incorporating adjustment, age restriction at NAS entry, and IPW for the KXRF subgroup (Figure 5D), now does not have either the collider stratification bias or conditioning on an intermediate bias problems identified in the original DAG (Figure 2).

Tests for linear trend across lead tertiles were computed by entering an ordinal variable corresponding to lead tertile as a continuous variable in the models. Statistical significance was evaluated with an alpha level of 0.05. All analyses were conducted using SAS, version 9.3 (SAS Institute Inc.), except for analyses incorporating penalized splines for the continuous lead term, which were completed using R, version 3.0.1 (R Core Team 2013).

## Results

At the time of bone lead measurement, the mean ± SD age of the 835 participants with bone lead data was 67 ± 7 years old. Most were well educated, and only 14% were current smokers (see Supplemental Material, Table S3). The health characteristics of those with a bone lead measurement and those without (either because they chose not to have it done or they were censored before the time of bone lead measurements) approximately 10 years before recruitment for the lead substudy (mean, 1983 ± 3 years) suggest that those without bone lead measurements are less healthy, although the differences are slight (Table 1). Notably, though, NAS men who provided bone lead measurements were more likely to be never smokers, suggesting generally better health habits overall.

**Table 1:** Characteristics at the time of first VA visit after 1980 (mean, 1983 ± 3 years) by age at NAS entry and participation in bone lead measurements among those who were alive at the time the bone lead analyses were started.

Characteristic	≤ 45 years of age at NAS entry *n *= 1,004		> 45 years of age at NAS entry *n *= 350	
	Bone lead measured (*n *= 637)	Bone lead not measured (*n *= 367)	Bone lead measured (*n *= 198)	Bone lead not measured (*n *= 152)
Smoking status [*n* (%)]
Never	167 (26.2)	74 (20.2)	78 (39.4)	43 (28.3)
Former	332 (52.1)	194 (52.9)	105 (53.0)	92 (60.5)
Current	137 (21.5)	99 (27.0)	14 (7.1)	16 (10.5)
Missing	1 (0.2)	0 (0)	1 (0.5)	1 (0.6)
Education [*n* (%)]
< High school	72 (11.3)	41 (11.2)	11 (5.6)	18 (11.8)
High school	217 (34.1)	130 (35.4)	67 (33.8)	57 (37.5)
Technical school	60 (9.4)	47 (12.8)	29 (14.7)	22 (14.5)
Some college	92 (14.4)	45 (12.3)	21 (10.6)	17 (11.2)
College graduate or more	172 (27.0)	88 (24.0)	63 (31.8)	28 (18.5)
Missing	24 (3.8)	16 (4.4)	7 (3.5)	10 (6.6)
History of heart disease [*n* (%)]	37 (5.8)	26 (7.1)	15 (7.6)	12 (7.9)
History of hypertension [*n* (%)]^*a*^	237 (37.2)	138 (37.6)	94 (47.5)	77 (50.7)
History of diuretic medications [*n* (%)]	73 (11.5)	39 (10.6)	28 (14.1)	31 (20.4)
History of cardiovascular medications [*n* (%)]	117 (18.4)	77 (21.0)	51 (25.8)	42 (27.6)
Age at visit (years) (mean ± SD)	53.3 ± 5.5	53.9 ± 6.4	64.4 ± 4.6	64.7 ± 4.2
Diastolic blood pressure (mmHg) (mean ± SD)	77.6 ± 8.7	79.0 ± 9.4	75.4 ± 8.2	75.8 ± 8.3
Systolic blood pressure (mmHg) (mean ± SD)	123.8 ± 14.7	125.7 ± 16.0	127.5 ± 16.4	129.3 ± 13.9
Total cholesterol (mg/dL) (mean ± SD)	241.1 ± 40.9	247.5 ± 45.5	242.6 ± 41.9	237.1 ± 41.9
Uric acid (mg/dL) (mean ± SD)	6.7 ± 1.3	6.6 ± 1.2	6.3 ± 1.1	6.5 ± 1.4
^***a***^Diagnosis, medications, or based on blood pressure.				

Results of our base model, restricted to white men and adjusting for education and smoking, suggested that there is a slight, but nonsignificant increase in the HR for all-cause, all cardiovascular, and ischemic heart disease mortality with increasing patella lead concentration tertile (Table 2, model 1). The effect estimates were materially unchanged with additional adjustment (Table 2, model 2), suggesting that our base adjustment variables reasonably controlled for SES or that bias from SES was minimal, although it cannot prove that we have not omitted some critical unmeasured (U) variable.

**Table 2:** Adjusted hazard ratios [HR (95% CI)] for all-cause, cardiovascular disease, and ischemic heart disease mortality, by tertile*^a^* of patella lead at baseline among either all white men in the Normative Aging Study (*n* = 835), or those ≤ 45 years old at NAS study entry (*n* = 637).

Model	Deaths	Tertile of patella Pb			*p*‑Trend
		1st (< 20 μg/g)	2nd (20–31 μg/g)	3rd (> 31 μg/g)	
Model 1: base model^*b*^ (*n* = 835)
All-cause mortality	235	Reference	1.23 (0.82, 1.85)	1.34 (0.90, 2.00)	0.16
All cardiovascular mortality	134	Reference	1.22 (0.71, 2.10)	1.46 (0.86, 2.48)	0.15
Ischemic heart disease mortality	61	Reference	1.73 (0.74, 4.07)	2.01 (0.86, 4.68)	0.12
Model 2: additional SES adjustment^*c*^ (*n* = 835)
All-cause mortality	235	Reference	1.16 (0.76, 1.79)	1.25 (0.83, 1.90)	0.30
All cardiovascular mortality	134	Reference	1.16 (0.65, 2.08)	1.45 (0.83, 2.53)	0.16
Ischemic heart disease mortality	61	Reference	1.96 (0.79, 4.88)	2.11 (0.87, 5.13)	0.13
Model 3: additional SES adjustment^*c*^ and restriction to ≤ 45 years old at NAS inception (*n* = 637)
All-cause mortality	135	Reference	1.30 (0.75, 2.26)	1.72 (0.98, 3.03)	0.05
All cardiovascular mortality	75	Reference	1.36 (0.63, 2.90)	2.23 (1.02, 4.84)	0.03
Ischemic heart disease mortality	35	Reference	2.74 (0.78, 9.63)	4.60 (1.26, 16.8)	0.02
Model 4: additional SES adjustment,^*c*^ restriction to ≤ 45 years old at NAS inception, and IPW (*n* = 637)
All-cause mortality	135	Reference	1.41 (0.86, 2.30)	1.86 (1.12, 3.09)	0.02
All cardiovascular mortality	75	Reference	1.53 (0.78, 2.99)	2.47 (1.23, 4.96)	0.009
Ischemic heart disease mortality	35	Reference	3.09 (1.01, 9.46)	5.20 (1.61, 16.8)	0.005
^***a***^Tertiles of patella lead are based on the distribution among NAS participants ≤ 45 years old at NAS entry. ^***b***^Model 1: adjusted for age at KXRF, age at KXRF squared, smoking (never/former/current and pack-years), and education. ^***c***^Additionally adjusted for occupation and salary at NAS entry, mother’s education and occupation, father’s education and occupation.					

*Restriction.* In analyses restricted to NAS participants who were ≤ 45 years old at entry into the NAS (*n* = 637), the magnitude of the association between the top tertile of patella lead concentration and mortality increased substantially, and the trends for all mortality categories were significant (Table 2, model 3).

*IPW.* Finally, using IPW to weight the maximally adjusted analysis among those ≤ 45 years of age at NAS recruitment further strengthened effect estimates (Table 2, model 4); analyses using weights truncated at the 1st and 99th percentiles were similar (see Supplemental Material, Table S4). The results of the base model analyses and of the additionally adjusted, restricted, and weighted analyses when treating patella lead concentration continuously and using splines are shown in Supplemental Material, Figures S1 and S2.

Analyses that considered blood lead or tibia lead concentration found no associations with mortality under any of the models. See Supplemental Material, Tables S5 and S6.

## Discussion

According to the assumptions about the causal structure detailed in our DAG, we applied methods to mitigate bias of the total exposure–health effect association through adjustment for age and SES-related variables, restriction to those ≤ 45 years of age at NAS entry, and use of IPW to account for nonparticipation after study entry. After applying these methods, we found that our patella lead–mortality effect estimates were substantially increased and consistently statistically significant; the effect estimate comparing those in the lowest tertile to the highest tertile of patella bone lead concentration increased 39% (HR = 1.34 to HR = 1.86) for all-cause mortality, 69% (HR = 1.46 to HR = 2.47) for all cardiovascular mortality, and 159% (HR = 2.01 to HR = 5.20) for ischemic heart disease mortality (Table 2).

Bias of the exposure–health effect association introduced by selection—both at cohort formation and later selection into subgroups within the formed cohort—can arise when these forms of selection are related to the outcome and the exposure under study. In the case of collider stratification bias related to selection into a study or substudy, this requires the exposure and outcome (or causes of the exposure or outcome, potentially including past exposure or outcome status) to determine selection (Greenland 2003). For many health studies it is likely that the outcome or its causes influence study enrollment and continued participation because health is an important predictor of participation, even absent cohort entry criteria that can produce the same phenomenon (Alonso et al. 2009; Mein et al. 2012). For environmental health studies in particular, exposures are often expected to be related to participation because such exposures are determined, in part, by socioeconomic status, which influences participation (de Graaf et al. 2000; Howe et al. 2013; Mein et al. 2012; Weuve 2013) and because current or future exposure is often highly correlated with past exposures, which may influence participation through their downstream consequences. Importantly, when selection is related to causes of the outcome that are also on the causal path between exposure and outcome, bias of the causal effect of the exposure on the health effect may arise from conditioning on an intermediate even if exposure is not otherwise related to selection. In our case, the selection process involved in creating the NAS led to conditioning on an intermediate, which produced a downward bias in the effect estimate for patella lead. Intuitively, if an intermediate factor between lead exposure and cardiovascular mortality is held fixed, then variation in lead exposure before that factor is irrelevant and can have no effect on the outcome through that intermediate factor because that factor cannot vary—everyone has the same level of it. Thus, any effect of Pb_0_ on cardiovascular mortality is blocked.

In our example, both of these sources of bias of exposure–health effect estimates can also be thought of as an issue of the depletion of susceptibles. Intuitively, the problem is that, on average, those people with high lead exposure who participate in the NAS KXRF examination may be a select sample of people who are much less sensitive to cardiovascular or other effects of lead exposure. If they were not less sensitive, then those effects of lead exposure would have prevented them from entering the NAS or participating in the KXRF substudy, either because they would be less inclined to participate due to poor health, or they would be excluded based on eligibility criteria for the NAS. In practice, analysis can be done only among those who were recruited into the study. Naïve analyses among this group must be interpreted as the association with lead exposure among those who entered the study, and we can argue that this is a group enriched with people who are not—or are less—sensitive to the cardiovascular effects of lead than what would be seen in the larger source population as a whole. Thus, even the association with lead exposure after entry into the study would be expected to be less than what one would get if the analysis was done on a population-representative group, although exactly how much less may be difficult to predict.

Given that for many environmental toxicants, exposures measured after study initiation are strongly correlated with exposures before study initiation, environmental studies may be highly susceptible to these biases, which would typically result in underestimation of the total adverse effects of the contaminants on the health outcome under study in the whole population from which one samples. When exposures after study initiation are not correlated with exposures before study initiation, the potential biases we describe are less likely. In pharmacoepidemiology studies, investigators often aim to avoid bias by considering only cohorts of “new users” of treatments. Studies of occupational exposures that enroll subjects when they start working can also avoid these problems because the workplace exposures occur only after study initiation. However, the ubiquitous nature of environmental exposures makes these issues highly problematic for environmental epidemiological studies. Similarly, studies of social or nutritional exposures would also likely suffer from these issues because those exposures also tend to be longstanding.

Although we believe that mitigation of effect estimate bias in our assumed causal structure accounts for our findings, alternate explanations are possible. For example, the change in results after restriction of the sample to those ≤ 45 years old at baseline, which we have labeled as attributable to mitigation of effect estimate bias, could also be explained by effect modification by age. That is, our results are also compatible with lead exposure at younger ages having a different effect on the risk of cardiovascular mortality than lead exposure at older ages. Specifically, if lead exposure at younger ages increases the risk of cardiovascular mortality, whereas lead exposure at older ages decreases—or at least does not increase—the risk of cardiovascular mortality, we would expect a difference in results similar to that which we found when restricting the sample to those ≤ 45 years old at NAS entry. However, from a biological perspective, a reduced effect of lead exposure at older ages seems unlikely; increasing age is often accompanied by increased vulnerability to stressors (Clegg et al. 2013).

The lack of association with tibia lead concentration in any analysis may imply that it is re-release of lead from bone at older ages that is most relevant for cardiovascular mortality than cumulative past exposure at earlier ages. Lead in patella is more mobilizable than lead in tibia, so it better reflects lead that can be re-introduced into circulation in later life as a result of bone reformation or bone loss than does lead in tibia, which better reflects long-term past exposures because of the very long half-life of lead in tibia bone (Wilker et al. 2011). The lack of association with blood lead concentration, though, suggests that the time window of relevance for these effects of lead re-released from bone is still a longer-term one because the half-life of lead in blood is on the order of a month, whereas that of lead in patella is on the order of years (Hu et al. 1998; Wilker et al. 2011). A few prior papers using National Health and Nutrition Examination Study (NHANES) data, however, did see associations between a single blood lead concentration measurement and mortality (Jemal et al. 2002; Lustberg and Silbergeld 2002; Menke et al. 2006; Schober et al. 2006). The different findings could relate to differences in ages of the study population, differences by race or sex, or—if the associations with blood lead were the result of correlation with bone lead—possibly because of more variability in lead exposure in the Boston, Massachusetts, area resulting in worse correlation with bone lead in our group than in NHANES.

This work has some limitations. Although we think our methods have mitigated bias of the exposure–health effect association, we acknowledge that they are unlikely to have completely eliminated it. For example, SES is a complex factor, and our adjustments may not have fully accounted for the aspects of SES that drive study participation. Similarly, restriction to those ≤ 45 years of age is unlikely to have completely eliminated bias of the exposure–health effect association, given that cardiovascular outcomes, though less common, still occur in that group. Finally, our IPW, as implemented, do not include lead measurements on nonparticipants and, therefore, assume that all effects of lead exposure on participation occur through measureable health effects or other conditions available in our data. Unmeasured confounding or incomplete control for confounding may remain a source of bias. Because our outcome was mortality, we ignored selection due to death and estimated our effect among the survivors at the time of the KXRF. Although IPW and other methods can be used to address selection due to death, the utility of such efforts is controversial (Chaix et al. 2012; Tchetgen Tchetgen et al. 2012; Weuve et al. 2012), and we refer the reader elsewhere (Andersen and Keiding 2012; Chaix et al. 2012; Kurland et al. 2009; Lau et al. 2009; Tchetgen Tchetgen 2014; Tchetgen Tchetgen et al. 2012; Varadhan et al. 2014; Weuve et al. 2012).

The problem of collider stratification bias in the setting of cohort studies is beginning to receive more attention. Several studies have shown that the magnitude of bias from loss to follow-up is potentially substantial, especially when SES is the exposure of interest (Howe et al. 2013; Weuve 2013; Weuve et al. 2012). Given the strong correlation between many environmental toxicants and SES, we would expect potentially large bias due to loss to follow-up to be possible and even expected because the combination of SES and factors related to many outcomes are likely to be a particularly strong predictor of participation, but are often difficult to measure. Similarly, bias from selective enrollment has been proposed as one potential explanation for a common pattern of association in studies of dementia risk, where associations with a risk factor (e.g., smoking, hypertension) suggest harm when participants are recruited and the risk factor is measured in mid-life, but protection when participants are recruited and the risk factor is measured in late life (Hernán et al. 2008; Power et al. 2011, 2013). The basis for this explanation posits that the most susceptible persons—those for whom the risk factor is most likely to result in dementia—are the most likely to be lost to follow-up, because both declining cognition and many of the risk factors of interest are known to influence attrition; as risk factor–related health effects and declining cognition are more likely manifest with advanced age, the problem worsens with older age of recruitment. However, these issues are not unique to health issues of older age. Birth cohorts, for example, can be affected in a similar manner by the fact that participants are (usually) selected on being live births. This can bias exposure–outcome effect estimates downward if the exposure under study is associated with reduced likelihood of conception or increased fetal loss (Hernán and Robins 2016). Our work suggests that selective enrollment in studies or substudies of environmental toxicants has the possibility to substantially bias results.

## Conclusions

Careful attention to the causal structure of one’s research study is critical to identifying potential biases and ways to mitigate them. Careful attention to factors that influence participation and loss to follow-up is critical, and may be especially important for studies of environmental risk factors. Recruitment of population-based samples and recruitment at earlier ages for all studies, including those of aging-related outcomes can help reduce these potential pitfalls by providing data necessary to address these issues analytically.

## Supplemental Material

(405 KB) PDF
