# Supplementary material for: Biased Exposure–Health Effect Estimates from Selection in Cohort Studies: Are Environmental Studies at Particular Risk?
Source: Environ Health Perspect. 2015 May 8;123(11):1113–22. doi: 10.1289/ehp.1408888 (PMC4629739; doi:10.1289/ehp.1408888)
Supplement: (405 KB) PDF [file ehp.1408888.s001.acco.pdf]

Environ Health Perspect
doi: 10.1289/ehp.1408888

Note to Readers: *EHP* strives to ensure that all journal content is accessible to all readers. However, some figures and Supplemental Material published in *EHP* articles may not conform to 508 standards due to the complexity of the information being presented. If you need assistance accessing journal content, please contact ehp508@niehs.nih.gov. Our staff will work with you to assess and meet your accessibility needs within 3 working days.

Supplemental Material

Biased Exposure–Health Effect Estimates from Selection in Cohort Studies: Are Environmental Studies at Particular Risk?

Marc G. Weisskopf, David Sparrow, Howard Hu, and Melinda C. Power

Table of Contents

Methods

Details of inverse probability weighting

Table S1. Variables used in forward selection logistic regression model to calculate inverse probability of attrition weights (IPW).

Table S2. Variables included in the final inverse probability of attrition weighting model.

Table S3. Characteristics at the time of bone lead measurement among all those with bone lead measurement (N=835).

Table S4. Fully adjusted[a] hazard ratios (95% confidence intervals) for all-cause, cardiovascular disease, and ischemic heart disease mortality, by tertile[b] of patella lead at baseline among white men in the Normative Aging 45 years old or younger at NAS study entry (N=637), and applying inverse probability of attrition (IPW) weights truncated at the 1st and 99th percentile of the distribution of the IPW weights.

Table S5. Adjusted hazard ratios (HR; 95% CI) for all-cause, cardiovascular disease, and ischemic heart disease mortality, by tertile[b] of blood lead at baseline among either all white men

in the Normative Aging Study (N=1,206), or those 45 years old or younger at NAS study entry (N=909).

Table S6. Adjusted hazard ratios (HR; 95% CI) for all-cause, cardiovascular disease, and ischemic heart disease mortality, by tertile of tibia lead at baseline among either all white men in the Normative Aging Study (N=834), or those 45 years old or younger at NAS study entry (N=636).

Figure S1. Nonlinear association between patella bone lead concentration and the log of HR (logHR) for all-cause, cardiovascular, and ischemic heart disease adjusted for age at KXRF, age at KXRF squared, smoking (never/former/current & packyears), and education among all white men (n=835) (Model 1: Base Model). The reference logHR=0 is at the mean of patella lead concentration. The estimates are indicated by the solid line and the 95% CIs by the dashed lines. The *P* values for significance of the nonlinear component for all-cause, cardiovascular, and ischemic heart disease mortality were 0.39, 0.54, and 0.64, respectively. Patella lead concentrations of all individual participants are indicated by short vertical lines on the x-axis..

Figure S2. Nonlinear association between patella bone lead concentration and the log of HR (logHR) for all-cause, cardiovascular, and ischemic heart disease adjusted for age at KXRF, age at KXRF squared, smoking (never/former/current & packyears), and education among white men 45 years old or younger at NAS entry (n=637) and with inverse probability weighting to weight the analyses to reflect the full group still alive at the time of KXRF (Model 4). The reference logHR=0 is at the mean of patella lead concentration. The estimates are indicated by the solid line and the 95% CIs by the dashed lines. The *P* values for significance of the nonlinear component for all-cause, cardiovascular, and ischemic heart disease mortality were 0.48, 0.91, and 0.28, respectively. Patella lead concentrations of all individual participants are indicated by short vertical lines on the x-axis.

Methods

Details of inverse probability weighting

We used a forward selection process to inform our final inverse probability of attrition weights (IPW) for the bone lead analyses. The variables considered in this process are shown in table S1. Our final model necessarily considered a subset of these, which are shown in table S2. The c-statistic from this model was 0.81. The unstabilized weights did not have very extreme values (see footnote, Table S2), and results for the fully adjusted models restricted to those 45 or younger at NAS entry using weights truncated at the 1st and 99th percentiles (i.e. assigning the 1st and 99th percentile weight to anyone with weights more extreme) were similar, only slightly weaker as expected (Table S4). IPW for blood lead analyses was done similarly to that for bone lead. The c-statistic for blood lead weighting models was 0.66.

doi: 10.1289/ehp.1408888

Table S1. Variables used in forward selection logistic regression model to calculate inverse probability of attrition weights (IPW).

Age, its square and cube
Date of birth
NAS visit number group (single ordinal variable 1-4 corresponding to NAS visits 1-4, 5, 6, 7+), its square and cube
NAS visit date
Education
Paternal education
Maternal education
Number of children
Years employed
White collar job (yes/no)
Marital status
Years smoked
Packyears smoked
Age started smoking
Consume 2 or more alcoholic drinks per day
Elevated blood pressure
Diagnosis since last NAS visit, ever diagnosis, age at first diagnosis, and years since first diagnosis for the following conditions: hypertension, diabetes, angina, cancer, heart disease, gout, myocardial infarction.
Current and ever use of the following medications: cholesterol, cardiovascular disease, diuretics, pain.
Blood laboratory variables. For the following variables we included a dichotomous cutoff variable for abnormal range value, the continuous variable (transformed if appropriate for its distribution) and its square: basophils, bilirubin, blood glucose, eosinophils, fasting glucose, hematocrit, hemoglobin, lymphocytes, monocytes, neutrophils, protein, red blood cell count, serum calcium, serum phosphorous, total cholesterol, triglycerides, uric acid, white blood cell count.
Additional health variables. For the following variables we included the variable (transformed if appropriate for its distribution) and its square: diastolic blood pressure, systolic blood pressure, body mass index, abdominal circumference, FEV1, FVC, FEV1/FVC ratio, maximum midexpiratory flow, salary at recruitment into NAS.
Interactions. Each of the following variables were included in interaction terms with age, square of age, and cube of age: education, NAS visit, NAS visit date, total cholesterol

Table S2. Variables included in the final inverse probability of attrition weighting model*.

Age and its square
NAS visit number group
NAS visit date, its square and its cube
Years since first diagnosis of diabetes
Ever diagnosis of angina
Current pain medication use
White collar job
Marital status
Abnormal fasting glucose
Abnormal hematocrit and its square
Blood protein and its square
Serum calcium
Diastolic blood pressure and its square
FVC
Body mass index
Abdominal circumference

* Final unstabilized censoring weights. Mean: 1.35; median: 1.26; min: 1.05; 1st percentile: 1.07; 5th percentile: 1.12; 10th percentile: 1.15; 90th percentile: 1.50; 95th percentile: 1.66; 99thpercentile: 2.50; max: 18.00.

Table S3. Characteristics at the time of bone lead measurement among all those with bone lead measurement (N=835).

Characteristic	n (%)
Smoking status	
Never	167 (20.0)
Former	548 (65.6)
Current	119 (14.3)
Missing	1 (0.1)
Education	
<High school	83 (9.9)
High school	284 (34.0)
Technical school	89 (10.7)
Some college	113 (13.5)
College graduate	141 (16.9)
Graduate school	94 (10.8)
Missing	31 (3.7)

Table S4. Fully adjusted[a] hazard ratios (95% confidence intervals) for all-cause, cardiovascular disease, and ischemic heart disease mortality, by tertile[b] of patella lead at baseline among white men in the Normative Aging 45 years old or younger at NAS study entry (N=637), and applying inverse probability of attrition (IPW) weights truncated at the 1st and 99th percentile of the distribution of the IPW weights.

		**Tertile of patella Pb**			
**Cause of death**	**Deaths**	**1st <20µg/g**	**2nd 20-31µg/g**	**3rd >31µg/g**	***p-trend***
All Cause Mortality	135	Ref	1.36 (0.83-2.23)	1.80 (1.09-2.98)	*0.02*
All Cardiovascular Mortality	75	Ref	1.48 (0.75-2.90)	2.37 (1.18-4.75)	*0.01*
Ischemic Heart Disease Mortality	35	Ref	3.08 (1.00-9.44)	5.08 (1.58-16.4)	*0.006*

[a] Adjusted for age at KXRF, age at KXRF squared, smoking (never/former/current & packyears), education, occupation and salary at NAS entry, mother's education and occupation, and father's education and occupation.

[b] Tertiles of patella lead are based on the distribution among NAS participants 45 years old or younger at NAS entry.

Table S5. Adjusted hazard ratios (HR; 95% CI) for all-cause, cardiovascular disease, and ischemic heart disease mortality, by tertile[b] of blood lead at baseline among either all white men in the Normative Aging Study (N=1,206), or those 45 years old or younger at NAS study entry (N=909).

		Tertile of blood Pb			
**Model**	**Deaths**	**1st <4µg/dL**	**2nd 4-6µg/dL**	**3rd >6µg/dL**	***p-trend***
**MODEL 1: Base Model[a] (N=1,206)**					
All Cause Mortality	320	Ref	0.96 (0.72-1.28)	0.99 (0.73-1.36)	*1.00*
All Cardiovascular Mortality	183	Ref	1.08 (0.73-1.60)	1.17 (0.77-1.78)	*0.47*
Ischemic Heart Disease Mortality	81	Ref	1.03 (0.57-1.86)	1.19 (0.64-2.22)	*0.56*
**MODEL 2: Additional SES Adjustment[c] (N=1,206)**					
All Cause Mortality	320	Ref	0.97 (0.72-1.31)	1.03 (0.74-1.43)	*0.84*
All Cardiovascular Mortality	183	Ref	1.10 (0.73-1.67)	1.28 (0.82-2.02)	*0.27*
Ischemic Heart Disease Mortality	81	Ref	1.01 (0.54-1.89)	1.08 (0.55-2.10)	*0.81*
**MODEL 3: Additional SES Adjustment[c] and Restriction To 45 years old or younger at NAS Inception (N=909)**					
All Cause Mortality	174	Ref	0.82 (0.55-1.23)	0.89 (0.58-1.38)	*0.68*
All Cardiovascular Mortality	94	Ref	0.98 (0.55-1.73)	1.20 (0.66-2.20)	*0.51*
Ischemic Heart Disease Mortality	45	Ref	0.81 (0.35-1.87)	0.87 (0.36-2.11)	*0.81*
**MODEL 4: Additional SES Adjustment[c], Restriction To 45 years old or younger at NAS Inception, and IPW (N=909)**					
All Cause Mortality	174	Ref	0.84 (0.59-1.20)	0.91 (0.62-1.34)	*0.70*
All Cardiovascular Mortality	94	Ref	1.01 (0.61-1.68)	1.25 (0.73-2.14)	*0.37*
Ischemic Heart Disease Mortality	45	Ref	0.83 (0.39-1.74)	0.90 (0.41-1.96)	*0.83*

[a] Model 1: Adjusted for age at blood draw, age at blood draw squared, smoking (never/former/current & packyears), and education.

[b] Tertiles of blood lead are based on the distribution among NAS participants 45 years old or younger at NAS entry.

[c] Additionally adjusted for occupation and salary at NAS entry, mother's education and occupation, father's education and occupation.

Table S6. Adjusted hazard ratios (HR; 95% CI) for all-cause, cardiovascular disease, and ischemic heart disease mortality, by tertile[b] of tibia lead at baseline among either all white men in the Normative Aging Study (N=834), or those 45 years old or younger at NAS study entry (N=636).

		Tertile of Tibia Pb			
**Model**	**Deaths**	**1st <15µg/g**	**2nd 15-22µg/g**	**3rd >22µg/g**	***p-trend***
**MODEL 1: Base Model[a] (N=834)**					
All Cause Mortality	234	Ref	0.95 (0.64-1.40)	1.02 (0.69-1.50)	*0.86*
All Cardiovascular Mortality	134	Ref	0.89 (0.54-1.49)	1.06 (0.64-1.75)	*0.72*
Ischemic Heart Disease Mortality	61	Ref	1.30 (0.62-2.74)	1.12 (0.51-2.44)	*0.87*
**MODEL 2: Additional SES Adjustment[c] (N=834)**					
All Cause Mortality	234	Ref	0.89 (0.59-1.33)	1.01 (0.67-1.51)	*0.85*
All Cardiovascular Mortality	134	Ref	0.83 (0.49-1.42)	1.03 (0.60-1.75)	*0.77*
Ischemic Heart Disease Mortality	61	Ref	1.30 (0.59-2.86)	1.10 (0.48-2.53)	*0.92*
**MODEL 3: Additional SES Adjustment[c] and Restriction To 45 years old or younger at NAS Inception (N=636)**					
All Cause Mortality	133	Ref	0.66 (0.39-1.10)	0.85 (0.50-1.44)	*0.65*
All Cardiovascular Mortality	74	Ref	0.49 (0.23-1.01)	0.99 (0.49-1.99)	*0.82*
Ischemic Heart Disease Mortality	35	Ref	1.59 (0.56-4.48)	1.49 (0.47-4.77)	*0.52*
**MODEL 4: Additional SES Adjustment[c], Restriction To 45 years old or younger at NAS Inception, and IPW (N=636)**					
All Cause Mortality	133	Ref	0.69 (0.44-1.09)	0.87 (0.54-1.40)	*0.68*
All Cardiovascular Mortality	74	Ref	0.54 (0.28-1.02)	1.04 (0.55-1.97)	*0.71*
Ischemic Heart Disease Mortality	35	Ref	1.68 (0.67-4.18)	1.59 (0.56-4.50)	*0.40*

[a] Model 1: Adjusted for age at KXRF, age at KXRF squared, smoking (never/former/current & packyears), and education.

[b] Tertiles of tibia lead are based on the distribution among NAS participants 45 years old or younger at NAS entry.

[c] Additionally adjusted for occupation and salary at NAS entry, mother's education and occupation, father's education and occupation.

Supplemental Material, Figure S1.

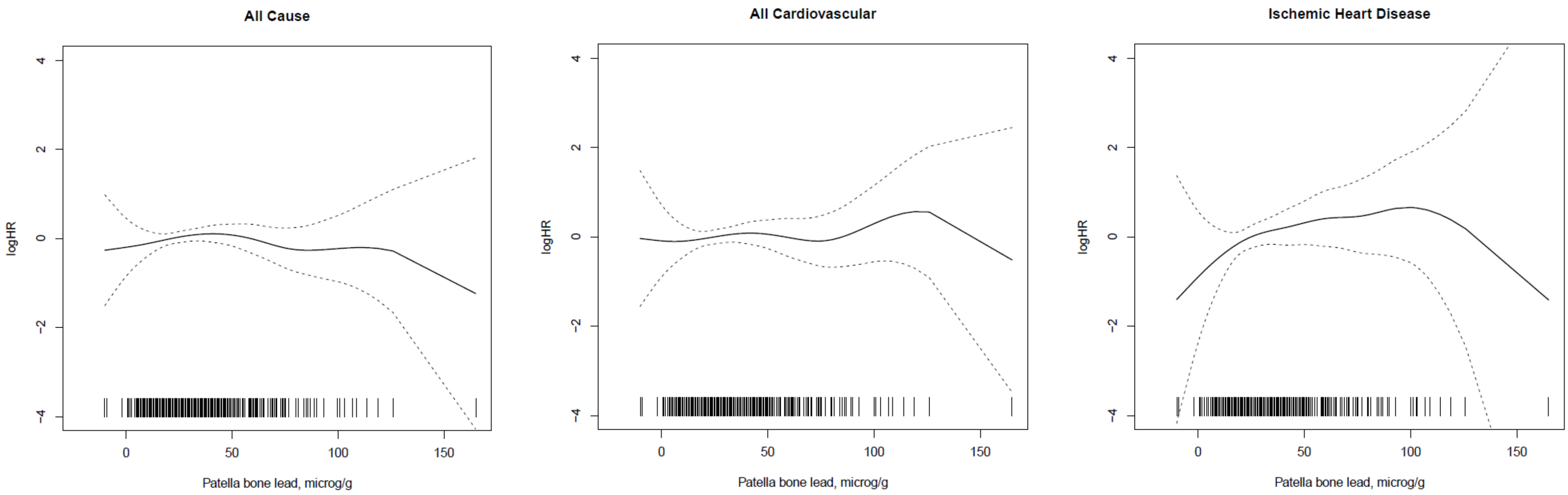

Figure S1. Nonlinear association between patella bone lead concentration and the log of HR (logHR) for all-cause, cardiovascular, and ischemic heart disease adjusted for age at KXRF, age at KXRF squared, smoking (never/former/current & packyears), and education among all white men (n=835) (Model 1: Base Model). The reference logHR=0 is at the mean of patella lead concentration. The estimates are indicated by the solid line and the 95% CIs by the dashed lines. The *P* values for significance of the nonlinear component for all-cause, cardiovascular, and ischemic heart disease mortality were 0.39, 0.54, and 0.64, respectively. Patella lead concentrations of all individual participants are indicated by short vertical lines on the x-axis.

Supplemental Material, Figure S2.

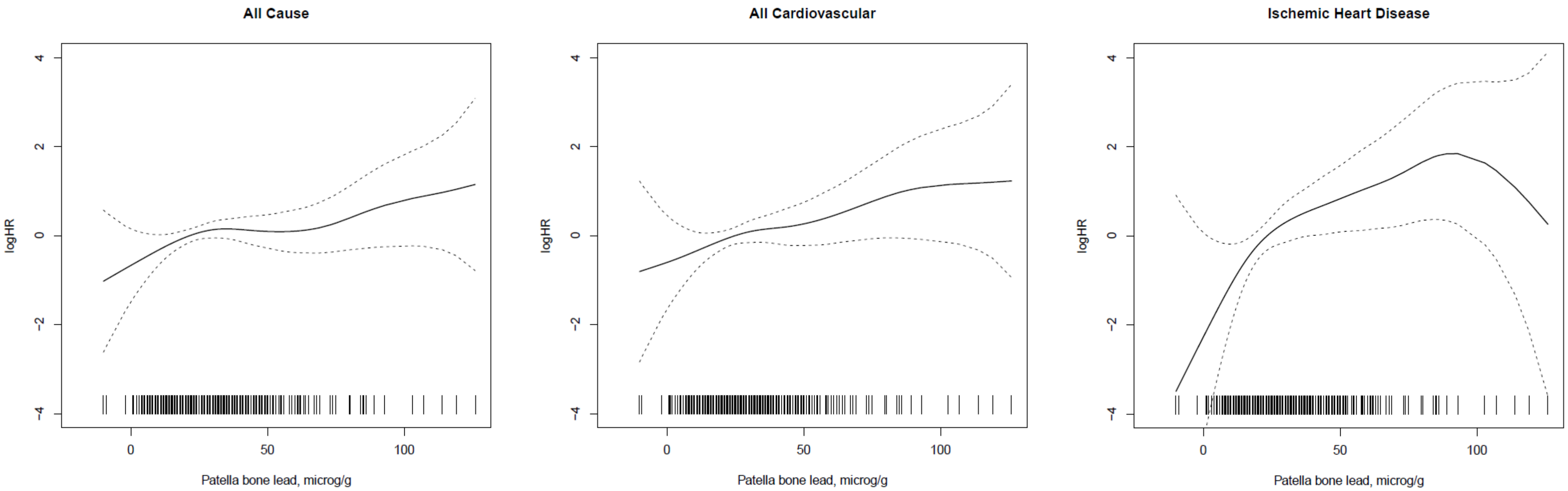

Figure S2. Nonlinear association between patella bone lead concentration and the log of HR (logHR) for all-cause, cardiovascular, and ischemic heart disease adjusted for age at KXRF, age at KXRF squared, smoking (never/former/current & packyears), and education among white men 45 years old or younger at NAS entry (n=637) and with inverse probability weighting to weight the analyses to reflect the full group still alive at the time of KXRF (Model 4). The reference logHR=0 is at the mean of patella lead concentration. The estimates are indicated by the solid line and the 95% CIs by the dashed lines. The *P* values for significance of the nonlinear component for all-cause, cardiovascular, and ischemic heart disease mortality were 0.48, 0.91, and 0.28, respectively. Patella lead concentrations of all individual participants are indicated by short vertical lines on the x-axis.
